# Validation and Comparison of Water Quality Products in Baltic Lakes Using Sentinel-2 MSI and Sentinel-3 OLCI Data

**DOI:** 10.3390/s20030742

**Published:** 2020-01-29

**Authors:** Tuuli Soomets, Kristi Uudeberg, Dainis Jakovels, Agris Brauns, Matiss Zagars, Tiit Kutser

**Affiliations:** 1 Institute for Environmental Solutions, Lidlauks, LV-4101 Priekuļu Parish, Latvia; dainis.jakovels@videsinstituts.lv (D.J.); agris.brauns@videsinstituts.lv (A.B.); matiss.zagars@videsinstituts.lv (M.Z.); 2 Tartu Observatory, University of Tartu, Observatooriumi 1, 61602 Tõravere, Estonia; kristi.uudeberg@ut.ee; 3 Estonian Marine Institute, University of Tartu, Mäealuse 14, 12618 Tallinn, Estonia; tiit.kutser@ut.ee

**Keywords:** water quality, optical properties, lakes, optically complex waters, remote sensing, Sentinel-2, Sentinel-3, MSI, OLCI, optical water types

## Abstract

Inland waters, including lakes, are one of the key points of the carbon cycle. Using remote sensing data in lake monitoring has advantages in both temporal and spatial coverage over traditional in-situ methods that are time consuming and expensive. In this study, we compared two sensors on different Copernicus satellites: Multispectral Instrument (MSI) on Sentinel-2 and Ocean and Land Color Instrument (OLCI) on Sentinel-3 to validate several processors and methods to derive water quality products with best performing atmospheric correction processor applied. For validation we used in-situ data from 49 sampling points across four different lakes, collected during 2018. Level-2 optical water quality products, such as chlorophyll-a and the total suspended matter concentrations, water transparency, and the absorption coefficient of the colored dissolved organic matter were compared against in-situ data. Along with the water quality products, the optical water types were obtained, because in lakes one-method-to-all approach is not working well due to the optical complexity of the inland waters. The dynamics of the optical water types of the two sensors were generally in agreement. In most cases, the band ratio algorithms for both sensors with optical water type guidance gave the best results. The best algorithms to obtain the Level-2 water quality products were different for MSI and OLCI. MSI always outperformed OLCI, with *R*^2^ 0.84–0.97 for different water quality products. Deriving the water quality parameters with optical water type classification should be the first step in estimating the ecological status of the lakes with remote sensing.

## 1. Introduction

The importance of inland water bodies has never been greater, because inland water, including lakes, are one of the key points of the carbon cycle and, therefore, local and regional climate [1,2]. Lakes are also economically important as a source of drinking water, and also provide fishing and recreation opportunities; therefore, the monitoring of the water quality has become a global concern. In Europe, for example, the Water Framework Directive [3] is aiming to achieve good water quality status in all lakes larger than 0.5 km^2^.

Lake water quality can be determined by its optical properties, as the concentrations of chlorophyll-a (Chl-a) and the total suspended matter (TSM), the absorption coefficient of colored dissolved organic matter (CDOM), and transparency of the water measured by Secchi disk (Secchi depth, SD). As lakes are known to be optically complex and more diverse than marine or ocean waters, these named optical properties can have larger temporal and spatial variation, depending on the changes in weather, biological composition, and physical attributes [4].

Phytoplankton, despite being microscopic, is the base of the lake ecosystem and has a big impact on water quality. The main pigment in the phytoplankton is Chl-a, it is one of the most well-known water quality parameters as it is used as a simple proxy of phytoplankton biomass globally [5]. Increasingly, lakes are affected by phytoplankton blooms driven by eutrophication caused largely by Cyanobacteria, witch, along with some other species of algae can produce potentially deadly toxins, which pose a health risk [6]. In addition, phytoplankton blooms are among the main influencers of water transparency and there is a negative correlation between those two parameters. Therefore, Chl-a is critical parameter to monitor the impacts of eutrophication, the ecological state of lakes, and the health risks from cyanobacteria blooms [6]. 

TSM contains organic and mineral suspended solids, which can originate from rivers or coasts, within the waterbodies, catchment areas, or be resuspended from the bottom of the lake. It can contain various substances: living and dead phytoplankton, humic substances, clay minerals, detritus, etc. [7]. As TSM has many components, it has been proven to be a challenge for remote sensing [8]. However, as TSM strongly influences the transparency of the water, and is related directly with many other variables (for example, turbidity, Secchi depth, water color), it is an important parameter to consider in lake management [8].

CDOM is a mixture of organic molecules produced during the decay of terrestrial vegetation, higher aquatic plants, phytoplankton, or bacteria. The estimates of CDOM are crucial in understanding the carbon cycle, as it is used as a proxy for the lake carbon content [9,10,11]. This parameter is also important in the context of the quality of drinking water as we need to be able to react quickly in the water treatment processes in the case of a sudden change in the CDOM concentrations in the water [12].

Secchi depth (SD) is an approximate evaluation of the water transparency with a Secchi disk; it is essentially a function of the reflection of the light from its surface. SD is strongly influenced by the three optically significant constituents named before (Chl-a, TSM, and CDOM) and generally, it corresponds to 10% of the surface light [13]. The relationship between SD and the light attenuation coefficient (*K*_d_) is known to be very good; therefore, in remote sensing, *K*_d_ is often used to derive SD. Also using different band ratios has shown good results in the SD retrieval in lakes [14,15]. Water transparency is, besides Chl-a, one of the most important parameters in the estimation of the ecological status of lakes [16].

There are two different Earth observation missions launched by the European Space Agency Copernicus program [17] that are suitable for lake studies. One of them is Sentinel-2, a land monitoring constellation of two satellites (A and B, launched in 2015 and 2017, respectively). Both have the Multispectral Instrument (MSI) onboard, which offers high-resolution optical imagery at 10 m, 20 m, and 60 m spatial resolution, depending on the spectral band. MSI samples in 13 spectral bands. This mission provides global coverage every 5 days [18]. Although, MSI has great spatial resolution that is suitable for smaller lakes, it lacks a band in critical wavelengths, such as the Chl-a absorption peak at 665 nm. The other suitable mission is Sentinel-3, which is also a constellation of two satellites (A and B, launched 2016 and 2018, respectively). Sentinel-3 has a medium resolution (300 m) Ocean and Land Color Instrument (OLCI) onboard for marine and land research. It has 21 spectral bands and provides global coverage (at the equator) every two days [19]. OLCI was built for water monitoring and has well placed spectral bands for that purpose. However, its rather low spatial resolution allows the study of only about 1000 of the largest lakes on Earth [20] out of 117 million [21]. 

Regardless of some of the technical issues, remote sensing has proven to be an effective method for monitoring water quality [4,22], having an advantage in temporal and spatial coverage compared with in-situ methods. Unfortunately, the possible synergies of using remote sensing products with in-situ data in national inland water monitoring programs remains largely unexploited, due to the cost assumptions, insufficient product accuracy, uncertain data continuity, and the lack of programmatic support (training, software, and management commitment) [22,23,24]. While Copernicus program data are free, with the program having data continuity plans for at least couple of decades, and the scientists are improving the satellite product quality constantly, the lack of programmatic support and lack of reliable water quality products for inland waters is still a concern to this day. Recent trends in deriving lake water quality products from larger areas or multiple lakes is the optical water type (OWT) guided approach [25,26,27,28,29], because, as said above, lake optical properties can vary largely in time and space. The pre-classification of each water pixel gives the opportunity to apply the most suitable algorithm for the given OWT. Based on the OWT guided approach mentioned above, Copernicus has launched a Global Land Service, that includes also 300 m resolution 10-day aggregated Lake Water Quality products (Chl-a and turbidity) and they are planning to release 100 m resolution products for the 200 largest lakes in Europe and Africa [30]. It is a good start for releasing inland water quality products but might not be enough for most of the national monitoring services, which need higher spatial resolution and more different water quality products (e.g., SD and TSM). Although, MSI was initially planned only for land monitoring, already several studies have shown its suitability for water monitoring [31,32,33,34]. Also, current studies can be considered as a step toward the development of the suitable algorithms and methods for inland water monitoring with MSI. 

Using the OWT guided approach was one of our goals in this study. Moreover, the main goal of this study was to find the best algorithms for estimating water quality parameters in lakes using both, MSI and OLCI data, when best performing atmospheric correction processor is used. In order to derive the water quality products, we first compared the atmospherically corrected reflectance spectra with in-situ reflectance spectra; secondly, we obtained and validated the OWTs from the reflectance spectra; and thirdly, we compared the performance of different water quality product algorithms.

## 2. Materials and Methods

### 2.1. In-Situ Data

**Figure 1:**
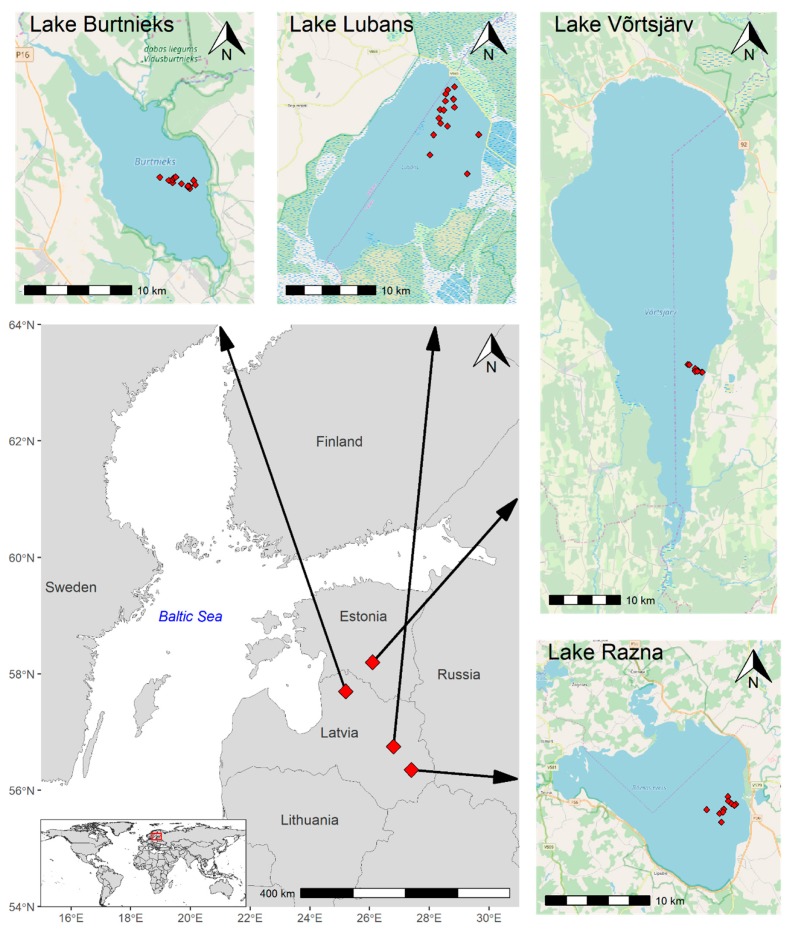
The location of the study lakes and their sampling points.

We gathered 49 samples from different sampling points using small boats from four optically different northern-boreal lakes and analyzed them in the laboratory during 10 field campaigns from April to November 2018 (Figure 1). Our four case study lakes were: Razna, Lubans, and Burtnieks in Latvia and Võrtsjärv in Estonia.

(1) Lake Razna is the second biggest lake in Latvia by area (57.6 km^2^) and the largest by water volume (0.46 km^3^) with a clear oligotrophic water [35]; where SD was measured at 3.2–6.6 m with median 5.5 m, the Chl-a was 1.4–10.9 mg m^−3^ with median 6.4 mg m^−3^, during 2018.
(2) Lake Lubans is a shallow, eutrophic, and macrophyte-dominated lake, where the water level is artificially regulated, in a way that the surface area of the lake can fluctuate from 25 to 100 km^2^ [35]. Our measured SD varied from 0.4–1.2 m with median 0.9 m, and the Chl-a was 8.6–63.1 mg m^−3^ with median 16.8 mg m^−3^.
(3) Lake Burtnieks is a shallow, eutrophic, and CDOM dominated northern Latvian lake with surface area 40.2 km^2^ [35], where the SD values are similar to Lake Lubans, 0.4–1.2 m with median 1 m, and the Chl-a was 6.7–117.1 mg m^−3^ with median 19.1 mg m^−3^.
(4) Lake Võrtsjärv is a second largest (270 km^2^) lake in Estonia. It is a large, shallow, very turbid, and eutrophic lake [36]. The measured SD was 0.5–0.1 m with a median 0.6 m, and the Chl-a was 22.2–63.8 mg m^−3^ with median 33.1 mg m^−3^ in 2018.

Field sampling campaigns included: collecting water samples from the surface layer; and measuring depth, temperature of the water and oxygen from about 0.5 m depth; as well as measuring water transparency with a Secchi disc. In addition, the reflectance spectra were measured above the surface (about 10 cm) using hand-held spectrometer PSR-3500 (Spectral Evolution Inc., Haverhill, MA, USA). The spectral range was 348–1000 nm and the spectral sampling interval approximately 1.5 nm.

Reflectance was calculated as the ratio of radiance from water to radiance from a white reference panel (99% Spectralon®, Labsphere Inc., North Sutton, NH, USA). In each sampling station, three measurements were made, and the average reflectance spectrum was calculated to represent measurement station reflectance; in this study, we use the term reflectance to mean the remote sensing reflectance. For MSI and OLCI analyses, in-situ measured reflectance spectra were calculated to the satellite bands using specific spectral response functions of the satellite sensor bands [29]. 

Laboratory analysis of water samples were carried out in Estonian Marine Institute, followed the methods of Lindell et al. [37] and they included:

- The spectrophotometric determination of Chl-a, where water sample aliquots were passed through 47 mm Whatman (Whatman plc., Maidstone, UK) glass microfiber filters with pore size 0.7 µm (GF/F), ethanol extracted, and then measured spectrophotometrically at 665 nm with a U-3010 laboratory spectrophotometer (Hitachi High-Technologies Corporation, Tokyo, Japan).
- Gravimetrically determined TSM, where pre-weighed and pre-combusted 47 mm Whatman GF/F glass microfiber filters were heated at 105 °C for 1 h.
- Determining the spectral absorption coefficients of CDOM using a U-3010 laboratory spectrophotometer (Hitachi High-Technologies Corporation) on samples prepared by successive filtrations through 47 mm Whatman GF/F filters and Nucleopore 0.2 µm filter paper.

### 2.2. Satellite Data

The MSI and OLCI cloud-free match-up images (±1-day included: up to three match-ups possible) were used. The ±1-day images were used, because the optical properties should not have rapid (overnight) changes in given lakes. This might be more likely in early spring (on the ice-melt), during autumn storms, or throughout other rapid weather change (heavy rains or winds). We were avoiding such weather conditions prior or during our field campaigns. We found 41 MSI match-ups for 35 different sampling points, from witch 6 sampling points had 2 match-up images; and 79 OLCI match-ups for 42 different sampling points, from witch 28 sampling points had more than one match-up images.

The MSI Level-1 images were downloaded from Copernicus Open Access Hub [38] and pre-processed by resampling the image to a 20 m resolution and applying the multi-sensor pixel identification tool (IdePix); only the “clear inland water” pixels were used. The atmospheric correction processor has a strong impact to the derived OWTs [29], so based on the recommendations of the previous studies [29,32], MSI images were further processed with Case-2 Regional CoastColour (C2RCC) and Case-2 Extreme Cases (C2X) processors [39] using scientific image processing toolbox called the Sentinel Application Platform (SNAP v 6.0) developed by Brockmann Consult, Array Systems Computing and C-S [40]. The salinity and temperature were modified in the processors according to in-situ data. In validation process we used a 3 × 3 pixel average instead of a single pixel value.

The OLCI Level-1 full resolution (300 × 300 m) images were downloaded from Copernicus Online Data Access [41]. The pre-processing of OLCI data were similar to the pre-processing of the MSI images, except no resampling was needed. For the atmospheric correction, there was only the C2RCC option available and not the C2X. A single pixel value was used in validation process. 2.3. Algorithms to retrieve the water quality products.

To obtain different optical properties, we used either the ready products from different atmospheric correction processors or band ratios using C2RCC, C2X, or top-of-atmosphere (TOA) reflectances that have shown good results previously in lakes or marine coastal areas [42]. Here too, we use term reflectance to mean the remote sensing reflectance. Details of the used sources are shown in Table 1. The *K*_d_490 was calculated using reflectances at 490, 560, and 705 nm (or 709.5 nm in the case of OLCI) [43].

In total, we tested 21 different options to derive Chl-a values from MSI data. Those were: C2RCC and C2X conc_chl [39]; Maximum Chlorophyll Index (MCI) Chl-a [45]; and six different band ratios from TOA, C2RCC, and C2X reflectances (Table 1). For OLCI we had somewhat less options, mostly due to the lack of C2X reflectances: Level 2 Neural Network (NN) Chl-a [44]; C2RCC conc_chl [39]; Maximum Peak Height/Chlorophyll-a (MPH/CHL) [46]; MCI Chl-a [45]; and six different band ratios from TOA and C2RCC reflectances (Table 1). 

We tested 14 different options to derive TSM values from MSI data. Those were: C2RCC and C2X conc_tsm [39]; and four different band ratios from TOA, C2RCC, and C2X reflectances (Table 1). For OLCI data we had nine different options: C2RCC conc_tsm [39]; and four different band ratios from TOA and C2RCC reflectances (Table 1). 

We tested 10 different options to derive CDOM values from MSI data. Those were: C2RCC and C2X iop_agelb [39]; and four different band ratios of C2RCC and C2X reflectances (Table 1). For OLCI we had five different options: C2RCC iop_agelb [39]; and four different band ratios of C2RCC reflectances (Table 1). 

We tested 21 different options to derive SD values from MSI data. Those were: C2RCC and C2X Kd_489 and Kd_z90max [39]; the *K*_d_490 algorithm from TOA, C2RCC, and C2X reflectances [43]; and in addition, a *K*_d_PAR algorithm [43] based on the above named *K*_d_490 products and a SD algorithm [60] based on *K*_d_PAR products. For OLCI, we had 12 different options: C2RCC Kd_489 and Kd_z90max [39]; *K*_d_490 algorithm [43] from TOA and C2RCC reflectances; and in addition, a *K*_d_PAR algorithm [43] based on the *K*_d_490 products and a SD algorithm [60] based on *K*_d_PAR products. 

**Figure A1:**
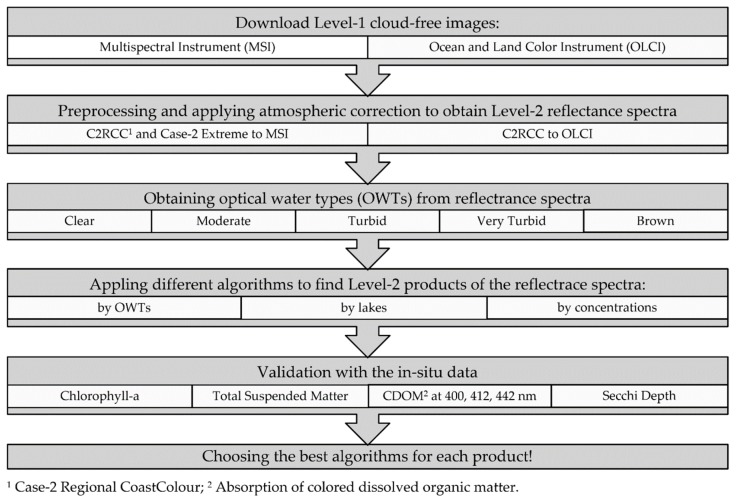
The flowchart of the different steps of the validation process.

For validating each parameter, we had tested grouping our results in three ways: by our four lakes; by the in-situ measured values (usually by low, medium, and high concentrations); and by the derived OWTs (Figure A1). We used always the OWTs from MSI C2X for the validation of the MSI water quality parameters, and OWTs derived from OLCI C2RCC for the validation of the OLCI water quality parameters. For the CDOM validation we compared the derived CDOM values with three different in-situ CDOM values: at 400 nm, 412 nm, and 442 nm, as the CDOM values from these three wavelengths are the most commonly used.

### 2.3. Classification of OWT 

The method by Uudeberg et al. [29] was applied for the classification of OWTs. This method classifies inland and coastal waters of boreal region into five OWTs, each with specific bio-optical conditions: (1) Clear OWT is water with the highest water transparency and the lowest optically active substance concentrations; (2) Moderate OWT is water with relatively higher concentrations of the optically significant constituents, but none of them dominate; (3) Turbid OWT is water where the TSM dominates; (4) Very Turbid OWT is water where the Chl-a dominates and this type is associated with blooms; (5) and Brown OWT is water where the CDOM dominates, and waters are dark and reddish to brown.

This method is based on reflectance spectrum key features, such as the location of spectral maximum, slopes, and amplitude. The OWT for each reflectance spectra of the pixel of the satellite image was determined by the maximum likelihood of individual spectra to reference spectra using a combination of spectral correlation similarity and modified spectral angle similarity. The OWT classification was applied to each OLCI and MSI satellite image pixel reflectance spectrum.

### 2.4. Statistical and Performance Measures Used in the Study 

In-situ measurement dataset was described using median values and range. Median is the middle value separating the greater and lesser halves of the data; and range shows the difference between the largest and smallest value. 

The performance of OWT results from different processors (atmospheric correction approaches) was evaluated using accuracy as a measure. Accuracy represents correctly predicted OWT type cases (expressed as percentage) in comparison to the reference dataset. Error refers to a percentage the derived value was not the same as in-situ.

The performance of water quality products (Chl-a, TSM, CDOM and SD) from different processors and band ratios was evaluated using two measures: determination coefficient (*R*^2^) and root mean squared error (RMSE). *R*^2^ is used to analyze how well observed in-situ values are predicted by the model based on the proportion of total variation of outcomes explained by the model; it is calculated using Equation (1). RMSE is a frequently used measure of differences between values observed in-situ and predicted by a model, it is calculated using Equation (2).

(1) $$R^{2}=1-\frac{\sum_{i=1}^{n}{\left(y_{i}-\hat{y}\right)}^{2}}{\sum_{i=1}^{n}{\left(y_{i}-\bar{y}\right)}^{2}},$$

(2) $$RMSE=\sqrt{\frac{1}{n}\sum_{i=1}^{n}{\left(y_{i}-\hat{y}\right)}^{2}},$$

where, $\hat{y}$ is the predicted value, *y* is the observed value and $\bar{y}$ is the mean value of observed *y* values.

## 3. Results

### 3.1. In-Situ Water Quality Parameters

The median and the range of measured optical properties from 49 sampling points across four lakes during 2018, are shown in Table 2. The water transparency decreased towards the summer and again increased in autumn, in all of the studied lakes. The average Secchi depth of the measurements was below 1 m, except in Lake Razna (5.3 m). Temperatures in the lakes reached over 26 degrees at the end of July 2018. The highest concentration of Chl-a was measured in Lake Burtnieks (117 mg m^−3^ on 23 Aug 2018) and the lowest value was measured in Lake Razna (1.4 mg m^−3^ on 28 May 2018). The Chl-a was the lowest by the end of the June after a steady decrease of the spring bloom in April. From July, Chl-a started to increase reaching its maximum by the end of August, in all of the lakes except lake Võrtsjärv, where the concentrations kept rising until mid-October. The seasonal variability of TSM followed the seasonal patterns of Chl-a. CDOM showed reasonably low seasonal variability and decreased in all the lakes from spring to autumn. 

### 3.2. Reflectance Spectra

**Figure 2:**
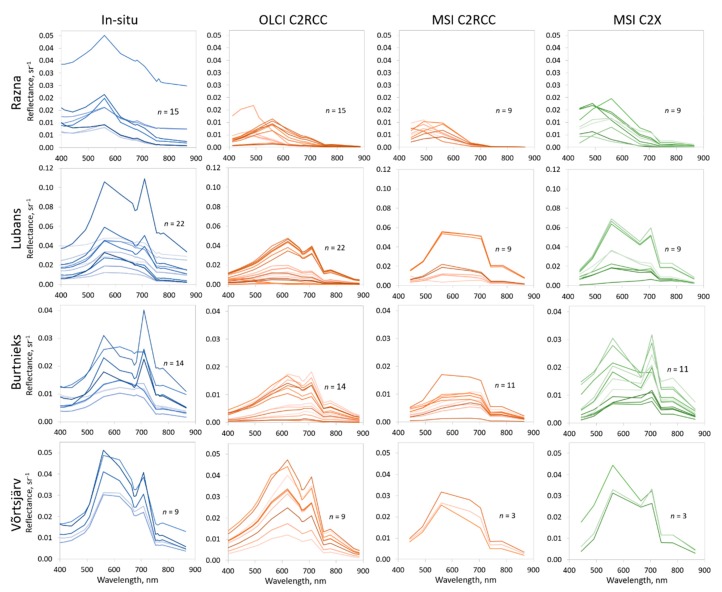
The reflectance spectra on different wavelengths measured in-situ and derived from satellites for each match up point. The in-situ reflectance spectra are calculated to the OLCI bands using OLCI spectral response functions; *n* marks the number of spectra.

The match-up in-situ and satellite’s sensors reflectance spectra for each lake are shown in Figure 2. The in-situ reflectance spectra are calculated to the OLCI bands used OLCI spectral response function. The MSI and OLCI reflectances are atmospherically corrected (C2RCC and C2X). 

**Figure A2:**
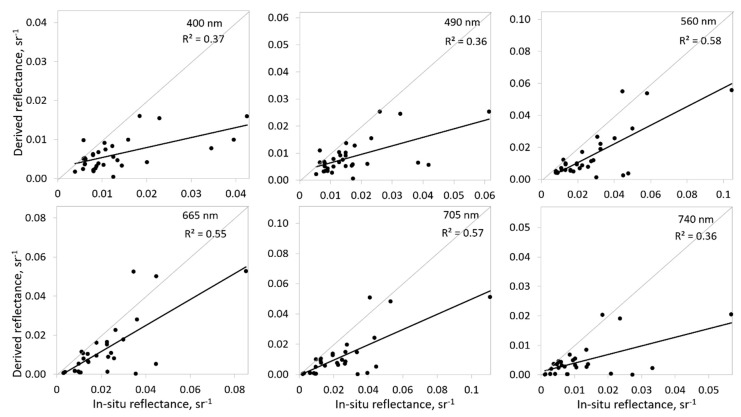
The comparison of the in-situ and MSI C2RCC spectral bands.

**Figure A3:**
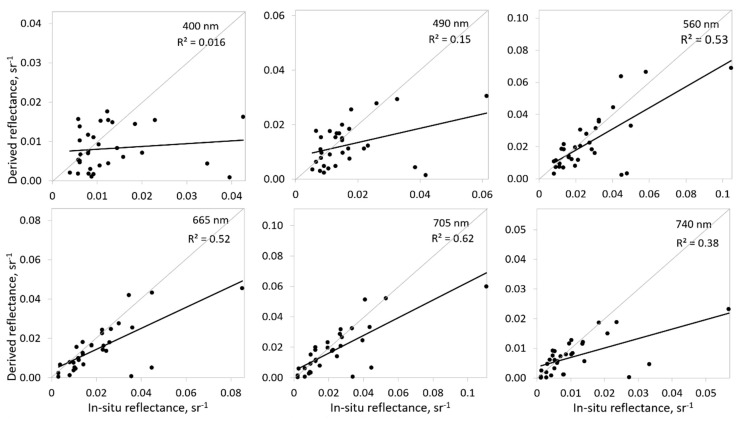
The comparison of the in-situ and MSI C2X spectral bands.

**Figure A4:**
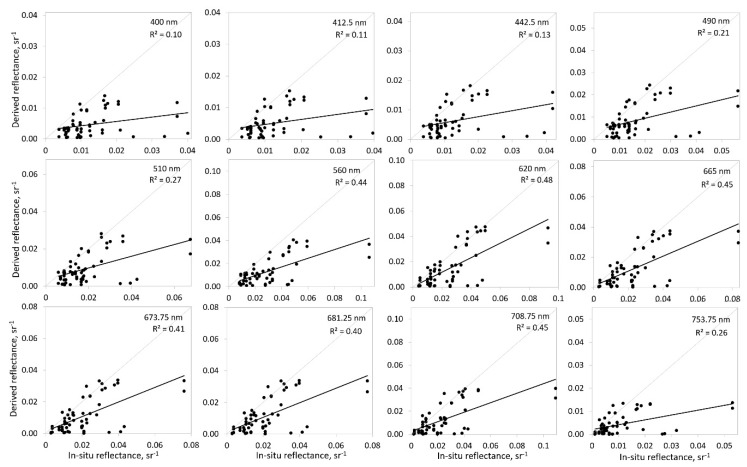
The comparison of the in-situ and OLCI C2RCC spectral bands.

Based on the comparison of the spectral bands (Figure A2, Figure A3 and Figure A4) and the general shape of the spectra, in most cases, the best performer was MSI C2X, as it replicates the shape of the spectra well. OLCI C2RCC also caught the shape of the spectra, thanks to more available bands in the visible range than the MSI. In both cases, MSI and OLCI, the C2RCC underestimated the reflectance spectra, especially on the shorter and longer wavelengths (Figure 2 and Figure A2, Figure A3 and Figure A4). 

### 3.3. Optical Water Types (OWTs)

**Figure 3:**
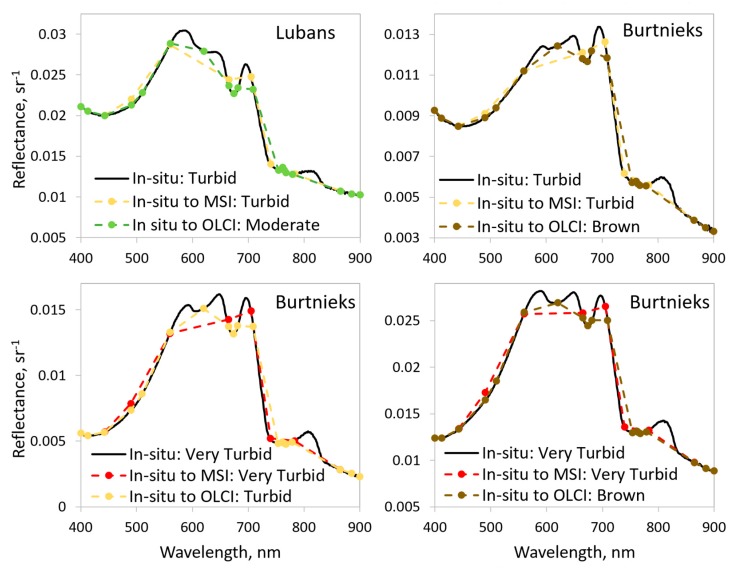
Four examples of obtaining different optical water types (OWTs) from in-situ hyperspectral reflectance spectra calculated to MSI and OLCI bands using specific spectral response functions.

Calculating the hyperspectral in-situ reflectance spectra to MSI bands did not change the obtained OWT. As MSI and OLCI have different bands for determining OWT, we first used in-situ data in the simulation to observe the possible differences on the MSI and OLCI bands to obtain the OWTs. All 38 match-up cases were determined correctly by using MSI bands. However, the calculation to the OLCI bands changed the obtained OWT in six cases. Half of them were from Turbid to Moderate, this and the other three possibilities are shown in Figure 3.

**Figure 4:**
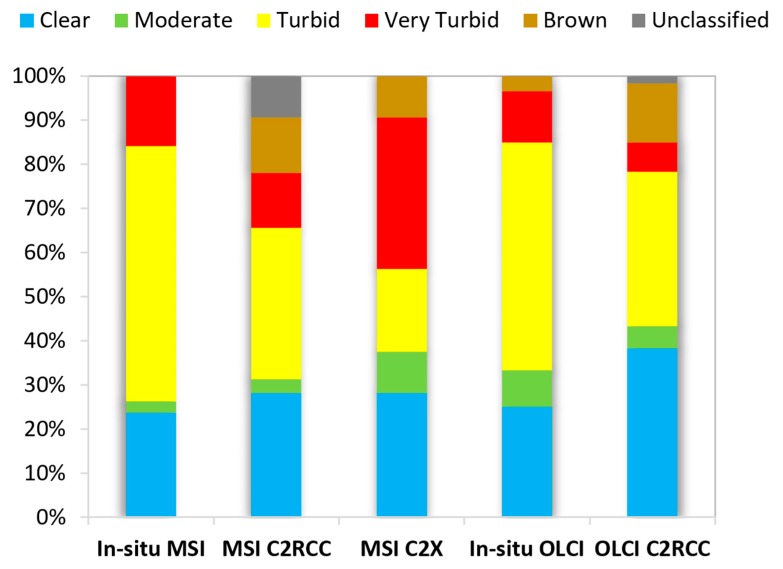
Distribution of the optical water type (OWT) classes. MSI in-situ, MSI C2RCC and C2X OWTs are for 32 match-ups. OLCI in-situ and OLCI C2RCC OWTs are for 60 match-ups.

We obtained several sets of OWTs: OWTs from in-situ reflectances calculated to MSI bands (MSI in-situ) and to OLCI bands (OLCI in-situ), MSI processed with C2RCC processor (MSI C2RCC) and C2X processor (MSI C2X), OLCI processed with C2RCC processor (OLCI C2RCC). We compared the MSI in-situ with MSI C2RCC and MSI C2X OWTs; and OLCI in-situ with OLCI C2RCC OWTs. The MSI C2X OWTs showed the highest potential in obtaining correct OWT (accuracy 72%, *R*^2^ = 0.81, and *n* = 32). MSI C2RCC accuracy was lower: 50%, *R*^2^ = 0.5. C2RCC of OLCI performed somewhat better: accuracy 65%, *R*^2^ = 0.49, *n* = 60. The detailed distribution of the obtained OWTs is shown in Figure 4.

**Figure 5:**
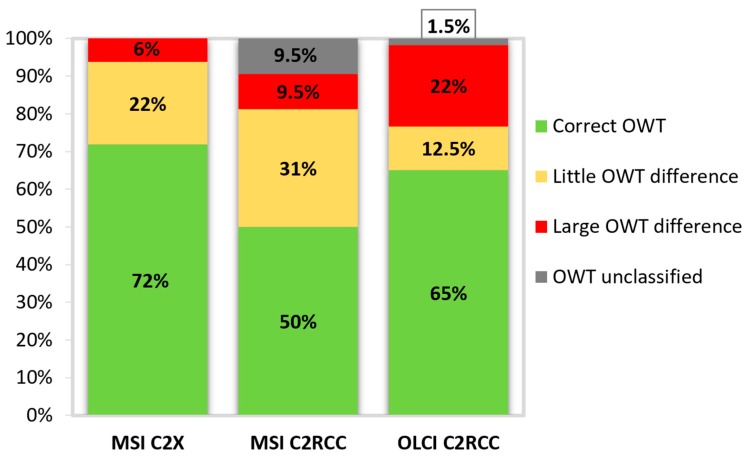
The distribution of the derived optical water types (OWTs) accuracy: same OWT as in-situ defined as correct OWT (green); neighboring OWT from in-situ defined as little OWT difference (yellow); more than neighboring OWT than in-situ defined as large OWT difference (red); and not classified to any OWT (grey).

To study the “magnitude of accuracy”, we looked at how serious the errors in determining the OWT were. The cause of misclassification may be the fact that the reflectance spectra were in a border of two OWT classes. Therefore, it might happen that slightly different reflectance spectra are classified differently. If the derived OWT classes were far from each other: Clear–Turbid, Clear–Very Turbid, Clear–Brown, or Turbid–Brown, this was defined as a large OWT difference, and all the other options (for example, Turbid–Very Turbid, or Clear–Moderate, etc.) were defined as a little OWT difference (Figure 5). 

The errors were rather small in MSI C2X derived OWTs (only 6% OWT are classified as large OWT difference from in-situ). It is also seen that in the case of OLCI the large error is much higher (22%), from which 60% of this error came from the misclassifying spectrum to the Clear OWT (MSI data did not have any of this kind of cases). MSI C2RCC has the largest percentage of unclassified cases (9.5%). 

### 3.4. Chlorophyll-a (Chl-a) 

**Figure 6:**
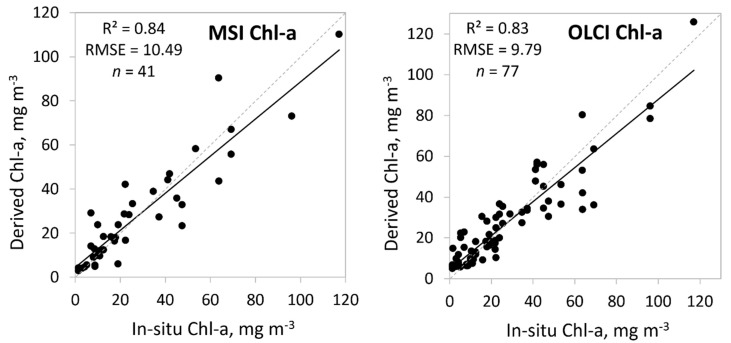
Correlation between in-situ and satellite derived chlorophyll-a (Chl-a, mg m^−3^) from MSI and OLCI based on the optical water types (OWTs). Also, *R*^2^, RMSE, and the number of samples (*n*) are given.

We compared 21 different methods to determine Chl-a values from MSI data with in-situ Chl-a values and 16 different methods for OLCI (Table 1). We searched for the best fits by sorting our data by lakes, in-situ Chl-a values, and by obtained OWTs. The best results for both, MSI and OLCI, gave different TOA band ratios for different OWTs using always either 705 nm or 709 nm in the band ratio. The overall results for both sensors, MSI and OLCI were quite similar: *R*^2^ = 0.84 for MSI and 0.83 for OLCI, the root mean square error (RMSE) was 10.5 mg m^−3^ for MSI and 9.8 mg m^−3^ for OLCI. The correlation of best performed TOA band ratios together are shown in Figure 6 and the details of the band ratios and their *R*^2^ are shown in Table 3. As seen from Figure 6 and Table 3, the low Chl-a values can be a challenge for sensors. 

### 3.5. Total Suspended Matter (TSM)

**Figure 7:**
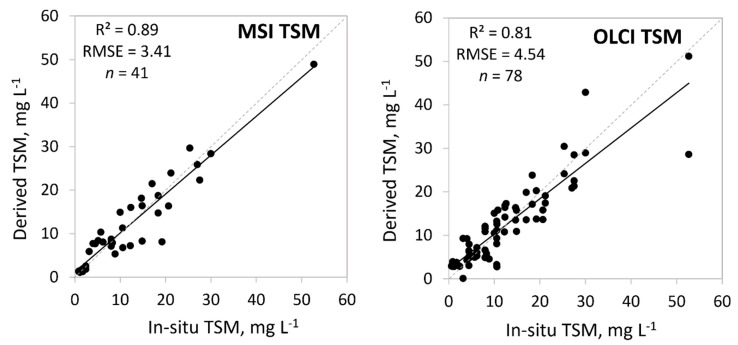
Correlation between in-situ and satellite derived total suspended matter (TSM, mg L^−1^) based on the optical water types (OWTs). Also, *R*^2^, RMSE, and the number of samples (*n*) are given.

We compared 14 different methods to determine TSM values from MSI data with in-situ TSM values and nine different methods for OLCI (Table 1). Here, for MSI, the C2RCC Level 2 product conc_tsm performed well for Moderate and Very Turbid OWTs, and generally, the C2RCC reflectances outperformed C2X. For OLCI different band ratios were showing the best results for different OWTs. The correlation of the best results with OWT guiding is shown in Figure 7 and the details are shown in Table 4. There was one case where the difference between the derived TSM and in-situ TSM was very large: in-situ 53 mg L^−1^, satellite derived 29 mg L^−1^ (Lake Lubans: in-situ Very Turbid OWT; OLCI Turbid OWT). This comes from different reflectance spectra, where the in-situ spectrum is 2.5 times higher than OLCI reflectance spectrum. MSI outperformed OLCI (*R*^2^ = 0.89 for MSI and 0.81 for OLCI). Again, the low concentrations were a challenge for OLCI. The result was slightly better when the data was grouped by the in-situ TSM values (Table 4).

### 3.6. Colored Dissolved Organic Matter (CDOM)

**Figure 8:**
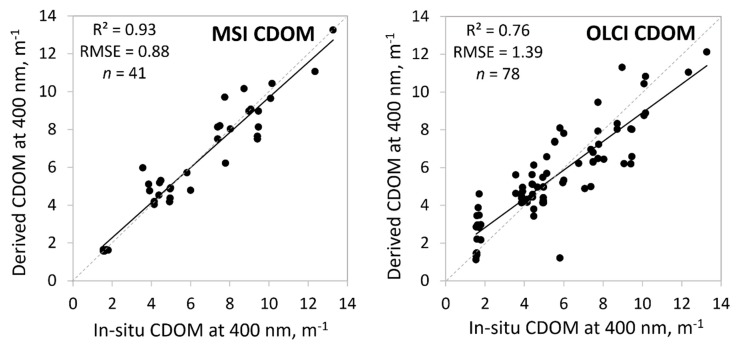
Correlation between in-situ and satellite derived colored dissolved organic matter (CDOM) at 400 nm based on the optical water types (OWTs). Also, *R*^2^, RMSE, and the number of samples (*n*) are given.

We compared 10 different methods to determine CDOM values from MSI data with in-situ CDOM values and five different methods for OLCI (Table 1). Yet again, we searched for the best fits by sorting our data by lakes, in-situ CDOM values, and by OWTs. The best results with the OWT guided approach gave a comparison with in-situ CDOM at 400 nm. MSI CDOM results were better than for OLCI (*R*^2^ = 0.91 for MSI and 0.76 for OLCI) (Figure 8). There is again one case standing out in the OLCI data: in-situ 5.8 m^−1^ and satellite derived 1.2 m^−1^ (Lake Lubans: in-situ Turbid OWT; OLCI Clear OWT). The in-situ and OLCI reflectance spectrum of this point was very different by its shape and magnitude (the in-situ spectrum was two times higher in the blue part of the spectrum, but 100 times higher in the red part). Although, the overall MSI result is very good then for the Clear OWT *R*^2^ was only 0.15 (Table 5). Here, as for TSM, for different OWTs the most suitable results might come from different options: the TOA, C2RCC, or C2X band ratios. 

### 3.7. Secchi Depth (SD)

**Figure 9:**
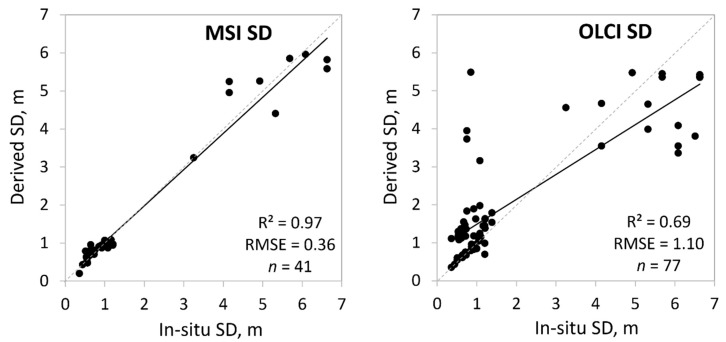
Correlation between in-situ and satellite derived Secchi depth (SD in m) based on the optical water types (OWTs). Also, *R*^2^, RMSE, and the number of samples (*n*) are given.

We compared 21 different methods to determine SD values from MSI data with in-situ SD values and 12 different methods for OLCI (Table 1). The OWT guided approach worked very well on MSI data (*R*^2^ = 0.97) (Figure 9), but OLCI performance was weaker (*R*^2^ = 0.69, RMSE = 1.1 m) (Figure 9). In all cases the Alikas et al. [43] algorithm to obtain the *K*_d_490 was the best choice (Table 6), however, the source was different (either TOA or C2X for MSI, and C2RCC or TOA for OLCI). 

## 4. Discussion

We used the C2RCC and C2X atmospheric correction processors for MSI, and C2RCC, for OLCI due to the results and recommendations of the previous studies [29,32,61]. The comparison of the in-situ and satellite sensors reflectance spectra for each lake revealed MSI C2X as the best performer in most cases. It was surprising that even in the oligotrophic lake like Razna with only Clear OWTs, the obtained reflectance spectra magnitude and shape were quite similar to the in-situ spectra with the MSI C2X processor (Figure 2). Hence, we assumed that also the derived OWTs were the most accurate based on MSI C2X spectra (accuracy 72%, *R*^2^ = 0.81). The OLCI and MSI C2RCC processed reflectance spectra showed large underestimation of the reflectance spectra. This also often missed the Chl-a absorption peak on or around 665 nm; the Chl-a peak in reflectance spectra of eutrophic waters can shift towards longer wavelengths with increasing biomass [53]. Lake Razna reflectance spectra demonstrate typical Clear OWT spectra; while Lake Burtnieks shows large peaks in the second Chl-a absorption maximum. 

The study of the MSI and OLCI spectral resolution impact to the classification of the OWT showed that MSI bands are very good for the detection of the OWTs. From 38 hyperspectral in-situ reflectance spectra calculated to MSI and OLCI, the bands showed a 100% match in the case of MSI, but only 85% matches in the case of OLCI bands. Although authors in [29] found both instruments equally accurate for determining the OWTs, in a current study, the OLCI bands might end up with different OWTs. Figure 3 shows some of these examples of how different placements of the spectral bands can lead to deriving different OWTs. Usually the derived OWTs are neighboring (Turbid and Very Turbid), however, there might be bigger differences (Turbid and Brown). Similarly, Uudeberg et al. [29] found that for 8% of Very Turbid OWTs, OLCI misclassified them as Turbid and 5% of Turbid OWTs were misclassified as Moderate. 

Due to of the slight differences in obtaining the OWTs for the MSI and OLCI bands, we used, in our OWT validation process, two different sets of in-situ OWTs: in-situ OWTs based on the MSI bands and based on the OLCI bands. It is shown that in the case of MSI the Clear OWT estimates are similar. But the other OWT classes might be more challenging (Figure 4). Our study of the accuracy of the OWT classification showed also that only 6% OWT are classified as “large OWT difference” from in-situ with MSI C2X (Figure 5). OLCI C2RCC largest errors come with the false-estimation of the Clear OWT class (in 14% of the cases from all the cases). These spectra might have been affected by the in-water vegetation, however, due to of the insufficient quality flagging and IdePix tool performance on OLCI data, they are not dismissed and are used as clear water pixels [61]. Without this misclassification of Clear OWT, the “large OWT difference” would be only 8%, instead of 22% (Figure 5). Based on these results, we decided that for the further water quality product validation the MSI C2X OWTs for MSI, and OLCI C2RCC OWTs for OLCI water quality products will be used. 

Overall, the best results for different inland water quality products were obtained using OWT classification and specific for each OWT band ratio (Figure 6, Figure 7, Figure 8 and Figure 9 and Table 3, Table 4, Table 5 and Table 6). Similar to Lins et al. [62], we also used the principle of OWT guiding, rather than other methods of clustering the data. Although, fitting the algorithms to the specific lake might slightly improve the results, the methods would not be applicable for all the lakes in our region. Similarly, with in-situ concentrations clustering, prior knowledge of the given lake would be needed, while with OWT guiding any prior knowledge of the lake or water pixel is not necessary. 

For the validation of the Chl-a we chose several algorithms proven to be successful in the given study area. For Chl-a, similarly to Toming et al. [31], the red and near-infrared (NIR) band ratios from TOA reflectance spectra were the most successful (Table 3). Overall MSI data were slightly better for obtaining the Chl-a in different OWTs (*R*^2^ = 0.84). The red and NIR band ratio for Brown OWT worked especially well for both satellites. In comparison with different algorithm performance for MSI, the MCI was performing even better, but only when the Chl-a was obtained for each lake separately (*R*^2^ = 0.91), so this approach demands the prior knowledge and adjusting the algorithm for each lake under the study. The results from OLCI were not as promising, with MCI grouped by lakes giving the overall average results, *R*^2^ = 0.61; *R*^2^ for MPH was even lower, 0.55. The only results slightly better than our selected OWT guided approach (*R*^2^ = 0.83) were the TOA band ratios guided by the in-situ Chl-a values (*R*^2^ = 0.85). The Clear OWT might be more challenging for sensors due to the typical Chl-a peaks that are not that pronounced at low Chl-a values, but surely the misclassified Clear OWT in the case of OLCI had part of the low correlation between the in-situ and OLCI derived Clear OWT Chl-a values (*R*^2^ = 0.34; Table 3).

The OWT-guided approach was the most successful in determining accurate TSM concentrations. In the case of MSI, the C2RCC Level 2 TSM product worked well for Moderate and Very Turbid OWTs. For the rest of the OWTs the 705 nm band value from different reflectance spectra was the best (Table 4). If considering that, previously, the validation of TSM has been very challenging due to its variable properties [8], the overall results of current study can be considered as very good (*R*^2^ = 0.89, RMSE = 3.4 mg L^−1^). In the case of OLCI, the results were somewhat weaker, overall *R*^2^ = 0.81, this was because, similarly to Chl-a, they failed to derive Clear OWT TSM concentrations (*R*^2^ = 0.21). For TSM, our results somewhat match with the results found in [42], where good performing TSM algorithms had bands from 700–710 nm.

Although the overall CDOM retrieval was very good for MSI (*R*^2^ = 0.93) with OWT guiding, the Clear OWT CDOM seemingly showed poor results (*R*^2^ = 0.15), while, in fact, the error to derive correct CDOM was less than 5% (RMSE only 0.07 m^−1^) (Table 5). This Clear OWT poor *R*^2^ value is caused by the small data range (in-situ 1.54–1.79 m^−1^; and MSI derived 1.57–1.65 m^−1^). For MSI, the best option was to use a two-band model, using red and green or blue bands, as also shown in some previous studies [9,31,42]. For OLCI the two-band model did not always work the best, and the three-band model offered the best fit, but still using red and green bands (Table 5). 

For water transparency, the use of Alikas et al.’s [43] method on MSI TOA (for Clear, Turbid, and Very Turbid) or C2X reflectances (for Brown) to derive *K*_d_490, gave the best results. Only for Moderate OWT, the best was to use C2X *K*_d_489 product. The overall *R*^2^ was very high (0.97) and RMSE was 0.36 m. The OLCI data was more challenging, due to some failings in turbid areas (in-situ SD < 1 m). However, it can also be observed that in the case of OLCI data for SD > 2 m the uncertainty of the derived SD increases; this could be related with the spatial resolution of OLCI sensor, signal strength at such depths, or turbidity. The overall RMSE was large for OLCI, 1.1 m (*R*^2^ = 0.69). Here the *K*_d_490 calculations are based on TOA reflectance bands for Very Turbid and Brown OWTs. For the other OWTs the atmospherically corrected reflectances worked the best. In many cases, the similar *K*_d_490 algorithm has been successful [16,63] even if they have not considered using the TOA reflectances. 

Overall the MSI seems to work better in lake water quality remote sensing than OLCI. The advantage for MSI might come from the C2X option, which shows good results in atmospheric correction and therefore the classification of the OWTs, and is, unfortunately, currently not available for OLCI. It is very important to retrieve accurate reflectance spectrum in order to obtain the correct OWT, when using the OWT guided approach for water quality retrieval. Therefore, the atmospheric correction should be chosen carefully. The MSI and especially OLCI C2RCC atmospheric correction over the lakes should be improved, because even a small error can cause significant changes in obtained Chl-a, TSM, CDOM, or SD [11]. The most challenging part of this study proved to be obtaining the Chl-a, TSM, and especially, SD for the Clear OWT from OLCI data. The challenges with data have been described before, mostly in coastal areas [11,42,64]. The issues with the retrieval of the water quality products for Clear OWTs might be due to several reasons. Firstly, in the case of low concentrations, the specific spectral shape for different optical properties might not be obvious and the spectra might miss the maximum absorption peaks necessary for the further calculations. Secondly, the errors in the classification of the OWTs, as a substantial proportion of the cases were falsely classified to Clear OWT, which comes either from the atmospheric correction issues over lakes with C2RCC [29,61] or from the poor clear water pixel masking of the processors and IdePix tool (especially for the OLCI data) [61]. 

Despite the relatively good overall accuracy of the remote sensing products, several issues still remain to be addressed in order to incorporate remote sensing into the national monitoring programs: the above-mentioned atmospheric correction of OLCI, spatial resolution of the OLCI that limits the use of it in smaller waterbodies; and lastly, the optical depth of the waters. The latter means that it is difficult to estimate water properties of the water column when the remote sensing signal comes primarily from the lake bottom or when water properties vary significantly in the water column [65,66,67]. The latter is not an issue in large lakes or seas where top tens of meters are uniformly mixed and the optical depth (from which the satellite signal originates) is smaller than the mixed layer depth. Smaller lakes, however, may have a quite sharp stratification of water properties that cannot be resolved with remote sensing. 

Future work should include studies on the spatial, vertical, and temporal variability of the water quality in the studied lakes; obtaining the ecological status of the lakes based on the Chl-a and SD products. Future work should also extend this method by offering the water quality products and ecological state estimation for all lakes in Latvia or even larger areas.

## 5. Conclusions

Using the OWT guided approach proved to be a very good method for estimating water quality parameters in lakes using both MSI and OLCI data. We used in-situ data from 49 sampling points across four different lakes collected during 2018 to validate MSI and OLCI Level 2 water quality products, such as Chl-a, TSM, CDOM, and SD. For processing the MSI and OLCI match-ups, we used C2RCC and C2X processors. MSI and OLCI C2RCC reflectance spectra mostly underestimated the in-situ reflectances, but MSI C2X were able to retrieve similar spectral shapes and often also the magnitude, even in clear waters. The MSI C2X reflectance spectra gave the most accurate OWTs (*R*^2^ = 0.8) and we used this for the water quality validation process. Our study shows that the used reflectance spectra have a large impact on the OWT guided approach to estimate the water quality products, as the obtained OWTs are directly dependent on the shape of the reflectance spectra. For the water quality products, the algorithms from different band ratios based (often TOA reflectance) on OWTs gave the best results for both satellites. The MSI outperformed OLCI every time. As there are more issues with OLCI (atmospheric correction, spatial resolution, and masking), we recommend using MSI for national monitoring with OWT guiding prior application of the algorithms for the estimation of the water quality or the ecological state of the lakes.

## Figures and Tables

**Table 1:** Used algorithms to retrieve the water quality products: chlorophyll-a (Chl-a), total suspended matter (TSM), absorption of colored dissolved organic matter (CDOM) at 400, 412, and 442 nm, Secchi depth (SD), and light attenuation coefficient over 400–700 nm (*K*_d_PAR). R in band ratio algorithms notes the reflectance at given wavelength.

Multispectral Instrument			Ocean and Land Color Instrument		
Algorithm	Water Quality Product	Reference	Algorithm	Water Quality Product	Reference
C2RCC ^1^	Chl-a, TSM, CDOM, SD	[39]	C2RCC ^1^	Chl-a, TSM, CDOM, SD	[39]
C2X ^2^	Chl-a, TSM, CDOM, SD	[39]	NN ^3^	Chl-a	[44]
MCI ^4^	Chl-a	[45]	MCI ^4^	Chl-a	[45]
			MPH/CHL ^5^	Chl-a	[46]
R705/R665 ^6^	Chl-a	[47,48]	R709/R674	Chl-a	[49]
R740/R705-R740/R665	Chl-a	[50]	(R665-R709)*R754	Chl-a	[51]
Log(R705/R665)	Chl-a	[52]	R709-(R665+R754)/2	Chl-a	[53]
R665/R705	Chl-a	[48]	R665/R709	Chl-a	[48]
R665/R740	Chl-a	[48]	R674/R709	Chl-a	[54]
R740/R665	Chl-a	[55]	R620*R681/R412	Chl-a	[56]
R620*R681/R510	TSM, CDOM	[57]	R620*R681/R510	TSM, CDOM	[57]
R783 – R740 + R865/2	TSM	[12]	R779 - R754 + R865/2	TSM	[12]
R705	TSM	[47,48]	R709	TSM	[49]
R705-R754	TSM	[58]	R709-R754	TSM	[58]
R665/R550	CDOM	[47]	R665/R550	CDOM	[47]
R665/R490	CDOM	[48]	R665/R490	CDOM	[48]
R560/R665	CDOM	[59]	R560/R665	CDOM	[59]
0.4349*K_d_490+0.3291	*K*_d_PAR	[43]	0.4349*K_d_490+0.3291	*K*_d_(PAR)	[43]
1.6941*K_d_PAR^−0,677^	SD	[60]	1.6941*K_d_PAR^−0,677^	SD	[60]

^1^ Case-2 Regional CoastColour; ^2^ Case-2 Extreme Cases; ^3^ Neural Network; ^4^ Maximum Chlorophyll Index; ^5^ Maximum Peak Height/Chlorophyll-*a*; ^6^ R denotes the reflectance and the particular number after R indicates the wavelength.

**Table 2:** The area and average depth (D), median and range in parenthesis of Secchi depth (SD), Chlorophyll-a (Chl-a), total suspended matter (TSM), and colored dissolved organic matter (CDOM) at 400 nm from four different lakes collected during April to November 2018. The *n* denotes the number of samples. Three out of four of the studied lakes are situated in Latvia (LV) and one in Estonia (EE).

Lake	*n*	Area km^2^	D m	SD m	Chl-a mg m^−3^	TSM mg L^−1^	CDOM m^−1^
Razna (LV)	10	57.6	7	5.5 (3.4)	6.35 (9.5)	1.7 (4.8)	1.6 (0.4)
Lubans (LV)	16	25-100	1.6	0.9 (1.0)	16.8 (58.3)	10.5 (48.3)	5.7 (3.9)
Burtnieks (LV)	13	40.2	2.4	1 (0.8)	19.1 (110.1)	8 (23.9)	9.5 (6.6)
Võrtsjärv (EE)	10	270	2.7	0.6 (0.5)	33.1 (41.7)	13.5 (17.8)	4.5 (3.5)
Total	49			0.9 (6.27)	18.9 (115.7)	8.9 (52.1)	5.5 (11.7)

**Table 3:** The best MSI and OLCI top-of-atmosphere (TOA) band ratios and their *R*^2^ and RMSE for chlorophyll-a (Chl-a) based on the optical water types (OWTs).

OWT	Algorithm	Formula	*R* ^2^	RMSE
**MSI**				
Clear	TOA R740/R705-R740/R665	y = −109.1x + 20.32	0.67	2.04
Moderate	TOA R665/R705	y = −105.3x + 140.6	0.72	6.15
Turbid	TOA R665/R705	y = 104.5x − 78.32	0.61	9.17
Very Turbid	TOA R740/R705-R740/R665	y = −368.5x + 39.1	0.73	14.33
Brown	TOA R705/R665	y = 293.5x − 263.4	0.90	14.26
**OLCI**				
Clear	TOA R709/R674	y = 44.75x − 32.78	0.34	4.74
Moderate	TOA (R665-R709)*R754	y = −21601x + 23.78	0.72	4.85
Turbid	TOA R709-(R665+R754)/2	y = 1552.3x + 21.03	0.43	13.92
Very Turbid	TOA R665/R709	y = −246.33x + 291.75	0.77	11.21
Brown	TOA R665/R709	y = −316.56x + 365.88	0.96	8.00

**Table 4:** The best MSI and OLCI band ratios and their *R*^2^ and RMSE for total suspended matter (TSM) based on the optical water types (OWTs).

OWT	Algorithm	Formula	*R* ^2^	RMSE
**MSI**				
Clear	TOA R705	y = 46.19x + 0.15	0.60	0.34
Moderate	C2RCC conc_tsm	y = −0.91x + 39.29	0.72	2.78
Turbid	C2RCC R705	y = 531x + 2.73	0.86	3.28
Very Turbid	C2RCC conc_tsm	y = 0.41x + 0.05	0.85	4.64
Brown	C2X R705	y = 1144.9x − 2.28	0.87	3.41
**OLCI**				
Clear	C2RCC R620*R681/R510	y = 267.97x + 2.81	0.21	3.07
Moderate	C2RCC R779 − R754 + R865/2	y = −13814x + 17.65	0.36	4.19
Turbid	TOA R620*R681/R510	y = 496.38x − 6.07	0.66	6.07
Very Turbid	TOA R779 − R754 + R865/2	y = 771.07x + 3.35	0.94	3.55
Brown	TOA R709	y = 529.77x − 16.26	0.78	3.53

**Table 5:** The best MSI and OLCI band ratios and their *R*^2^ and RMSE for colored dissolved organic matter (CDOM) at 400 nm based on the optical water types (OWTs).

OWT	Algorithm	Formula	*R* ^2^	RMSE
**MSI**				
Clear	C2RCC R665/R490	y = 0.26x + 1.56	0.15	0.07
Moderate	TOA R665/R490	y = 81.28x^2^ − 80.38x + 23.91	0.91	0.10
Turbid	C2X R665/R560	y = 21.31x − 9.67	0.71	0.95
Very Turbid	TOA R665/R490	y = 148.99x^2^ − 203.38x + 73.33	0.73	1.28
Brown	C2RCC R665/R490	y = 6.43x^2^ − 19.19x + 20.27	1.00	0.001
**OLCI**				
Clear	C2RCC R665/R550	y = 7.035x + 0.34	0.70	1.50
Moderate	C2RCC R665/R490	y = 9.4e^−0.67x^	0.55	0.23
Turbid	TOA R620*R681/R510	y = 1618.6x^2^ − 225.9x + 12.02	0.34	1.47
Very Turbid	TOA R620*R681/R510	y = 1.97x^−0.4^	0.64	1.04
Brown	C2RCC R665/R490	y = 8.83x^2^ − 29.82x + 33.17	0.51	1.41

**Table 6:** The best MSI and OLCI band ratios and their *R*^2^ and RMSE for Secchi depth (SD) based on the optical water types (OWTs).

OWT	Algorithm	Formula	*R* ^2^	RMSE
**MSI**				
Clear	TOA *K*_d_490 ^1^	y = 2.21x^−1.42^	0.63	0.71
Moderate	C2X *K*_d_489	y = −0.18x + 1.44	0.97	0.03
Turbid	TOA *K*_d_490 ^1^	y = 0.7x^−0.9^	0.68	0.15
Very Turbid	TOA *K*_d_490 ^1^	y = 0.59x^−1.56^	0.78	0.14
Brown	C2X *K*_d_490 ^1^	y = 0.35x − 1.23	0.96	0.06
**OLCI**				
Clear	C2RCC *K*_d_490 ^1^	y = 0.37x^2^ − 2.7x + 5.63	0.48	1.70
Moderate	C2RCC *K*_d_490 ^1^	y = 2.98x^2^ − 8.32x + 6.48	0.80	0.40
Turbid	C2RCC *K*_d_489	y = 0.17x^2^ − 1.54x + 4.07	0.67	0.65
Very Turbid	TOA *K*_d_490 ^1^	y = 0.5x^−1.40^	0.95	0.03
Brown	TOA *K*_d_490 ^1^	y = 0.54x^−1.25^	0.87	0.09

^1^ Light attenuation coefficient at 490 nm (*K*_d_490) was calculated using reflectances at 490, 560, and 705 nm (or 709.5 nm in the case of OLCI) [43].

## References

[1] Williamson C.E., Saros J.E., Vincent W.F., Smol J.P. (2009). Lakes and reservoirs as sentinels, integrators, and regulators of climate change. Limnol. Oceanogr..

[2] Tranvik L.J., Downing J.A., Cotner J.B., Loiselle S.A., Striegl R.G., Ballatore T.J., Dillon P., Finlay K., Fortino K., Knoll L.B. (2009). Lakes and impoundments as regulators of carbon cycling and climate. Limnol. Oceanogr..

[3] The European Parliament, The Council of the European Union (2000). WFD Directive 2000/60/EC of the European Parliament and of the Council of 23 October 2000 establishing a framework for Community action in the field of water policy. Off. J. Eur. Parliam..

[4] Ogashawara I., Mishra D.R., Gitelson A.A., Mishra D.R., Ogashawara I., Gitelson A.A. (2017). Remote Sensing of Inland Waters: Background and Current State-of-the-Art. Bio-Optical Modeling and Remote Sensing of Inland Waters.

[5] Codd G.A. (2000). Cyanobacterial toxins, the perception of water quality, and the prioritization of eutrophication control. Ecol. Eng..

[6] Matthews M.W., Mishra D.R., Ogashawara I., Gitelson A.A. (2017). Bio-optical Modeling of Phytoplankton Chlorophyll-a. Bio-Optical Modeling and Remote Sensing of Inland Waters.

[7] Gustafsson Ö., Gschwend P.M. (1997). Aquatic colloids: Concepts, definitions, and current challenges. Limnol. Oceanogr..

[8] Giardino C., Bresciani M., Braga F., Cazzaniga I., Keukelaere L., Knaeps E., Brando V.E., Mishra D.R., Ogashawara I., Gitelson A.A. (2017). Bio-optical Modeling of Total Suspended Solids. Bio-Optical Modeling and Remote Sensing of Inland Waters.

[9] Kutser T., Pierson D.C., Kallio K.Y., Reinart A., Sobek S. (2005). Mapping lake CDOM by satellite remote sensing. Remote Sens. Environ..

[10] Kutser T., Verpoorter C., Paavel B., Tranvik L.J. (2015). Estimating lake carbon fractions from remote sensing data. Remote Sens. Environ..

[11] Kutser T., Koponen S., Kallio K., Fincke T., Paavel B., Mishra D.R., Ogashawara I., Gitelson A.A. (2017). Bio-optical Modeling of Colored Dissolved Organic Matter. Bio-Optical Modeling and Remote Sensing of Inland Waters.

[12] Kutser T., Paavel B., Verpoorter C., Ligi M., Soomets T., Toming K., Casal G. (2016). Remote Sensing of Black Lakes and Using 810 nm Reflectance Peak for Retrieving Water Quality Parameters of Optically Complex Waters. Remote Sens..

[13] Wetzel R.G. (2001). Limnology. Lake and River Ecosystems.

[14] Arst H., Kutser T. (1994). Data processing and Interpretation of the Radiance Factor Measurements. Polar Res..

[15] Olmanson L.G., Bauer M.E., Brezonik P.L. (2008). A 20-year Landsat water clarity census of Minnesota’s 10,000 lakes. Remote Sens. Environ..

[16] Alikas K., Krazer S. (2017). Improved retrieval of Secchi depth for optically-complex waters using remote sensing data. Ecol. Indic..

[17] Copernicus. www.copernicus.eu.

[18] ESA Sentinel Online, Sentinel-2. sentinel.esa.int/web/sentinel/missions/sentinel-2.

[19] ESA Sentinel Online, Sentinel-3. sentinel.esa.int/web/sentinel/missions/sentinel-3.

[20] Globolakes Global Observatory of Lake Responses to Environmental Change. www.globolakes.ac.uk.

[21] Verpoorter C., Kutser T., Seekell D., Tranvik L. (2014). A Global Inventory of Lakes Based on High-Resolution Satellite Imagery. Geophys. Res. Let..

[22] Dörnhöfer K., Oppelt N. (2016). Remote sensing for lake research and monitoring-Recent advances. Ecol. Indic..

[23] Schaeffer B.A., Schaeffer K.G., Keith D.J., Lunetta R.S., Conmy R., Gould R.W. (2013). Barriers to adopting satellite remote sensing for water quality management. Int. J. Remote Sens..

[24] Palmer S.C., Kutser T., Hunter P.D. (2015). Remote sensing of inland waters: Challenges, progress and future directions. Remote Sens. Environ..

[25] Le C., Li Y., Zha Y., Sun D., Huang C., Zhang H. (2011). Remote estimation of chlorophyll a in optically complex waters based on optical classification. Remote Sens. Environ..

[26] Shi K., Li Y., Zhang Y., Li L., Lv H., Song K. (2014). Classification of inland waters based on bio-optical properties. J. Sel. Top. Appl. Earth Obs. Remote Sens..

[27] Shen Q., Li J., Zhang F., Sun X., Li J., Li W., Zhang B. (2015). Classification of several optically complex waters in China using in situ remote sensing reflectance. Remote Sens..

[28] Spyrakos E., O’Donnell R., Hunter P., Miller C., Scott M., Simis S., Neil C., Barbosa C., Binding C., Bradt S. (2018). Optical types of inland and coastal waters. Limnol. Oceanogr..

[29] Uudeberg K., Ansko I., Põru G., Ansper A., Reinart A. (2019). Using Optical Water Types to Monitor Changes in Optically Complex Inland and Coastal Waters. Remote Sens..

[30] Copernicus Global Land Service. land.copernicus.eu/global/products/lwq.

[31] Toming K., Kutser T., Laas A., Sepp M., Paavel B., Nõges T. (2016). First Experiences in Mapping Lake Water Quality Parameters with Sentinel-2 MSI Imagery. Remote Sens..

[32] Ansper A., Alikas K. (2019). Retrieval of Chlorophyll a from Sentinel-2 MSI Data for the European Union Water Framework Directive Reporting Purposes. Remote Sens..

[33] Molkov A.A., Fedorov S.V., Pelevin V.V., Korchemkina E.N. (2019). Regional Models for High-Resolution Retrieval of Chlorophyll a and TSM Concentrations in the Gorky Reservoir by Sentinel-2 Imagery. Remote Sens..

[34] Zeng C., Binding C. (2019). The Effect of Mineral Sediments on Satellite Chlorophyll-a Retrievals from Line-Height Algorithms Using Red and Near-Infrared Bands. Remote Sens..

[35] Ezeri.lv. www.ezeri.lv.

[36] Mäemets A. (1977). Eesti NSV Järved ja Nende Kaitse.

[37] Lindell T., Pierson D., Premazzi G., Zilioli E., European Commission, Joint Research Centre (European Commission) (1999). Manual for Monitoring European Lakes Using Remote Sensing Techniques.

[38] Copernicus Open Access Hub. Scihub.copernicus.eu.

[39] Brockmann C., Doerffer R., Peters M., Kerstin S., Embacher S., Ruescas A. (2016). Evolution of the C2RCC neural network for Sentinel 2 and 3 for the retrieval of ocean colour products in normal and extreme optically complex waters. Living Planet Symposium.

[40] Zuhlke M., Fomferra N., Brockmann C., Peters M., Veci L., Malik J., Regner P. (2015). SNAP (sentinel application platform) and the ESA sentinel 3 toolbox. Sentin.-3 Sci. Workshop.

[41] Copernicus Online Data Access. Coda.eumetsat.int.

[42] Ligi M., Kutser T., Kallio K., Attila J., Koponen S., Paavel B., Soomets T., Reinart A. (2017). Testing the performance of empirical remote sensing algorithms in the Baltic Sea waters with modelled and in situ reflectance data. Oceanologia.

[43] Alikas K., Kratzer S., Reinart A., Kauer T., Paavel B. (2015). Robust remote sensing algorithms to derive diffuse attenuation coefficient for lakes and coastal waters. Limnol. Oceanogr. Methods.

[44] Doerffer R., Schiller H. (2007). The MERIS Case 2 water algorithm. Int. J. Remote Sens..

[45] Gower J.F.R., Doerffer R., Borstad G.A. (1999). Interpretation of the 685nm peak in water-leaving radiance spectra in terms of fluorescence, absorption and scattering, and its observation by MERIS. Int. J. Remote Sens..

[46] Matthews M.W., Bernard S., Robertson L. (2012). An algorithm for detecting trophic status (chlorophyll-a), cyanobacterial-dominance, surface scums and floating vegetation in inland and coastal waters. Remote Sens. Environ..

[47] Ammenberg P., Flink P., Lindell T., Pierson D., Strombeck N. (2002). Bio-optical modelling combined with remote sensing to assess water quality. Int. J. Remote Sens..

[48] Koponen S., Attila J., Pulliainen J., Kallio K., Pyhälahti T., Lindfors A., Rasmus K., Hallikainen M. (2007). A case study of airborne and satellite remote sensing of a spring bloom event in the Gulf of Finland. Cont. Shelf Res..

[49] Kallio K., Kutser T., Hannonen T., Koponen S., Pulliainen J., Vepsäläinen J., Pyhälahti T. (2001). Retrieval of water quality from airborne imaging spectrometry of various lake types in different seasons. Sci. Total Environ..

[50] Zimba P.V., Gitelson A. (2006). Remote estimation of chlorophyll concentration in hyper-eutrophic aquatic systems: Model tuning and accuracy optimization. Aquaculture.

[51] Gitelson A., Dall’Olmo G., Moses W., Rundquist D., Barrow T., Fisher T., Gurlin D., Holz J. (2008). A simple semi-analytical model for remote estimation of chlorophyll-a in turbid waters: Validation. Rem. Sens. Environ..

[52] Hunter P., Tyler A.N., Willby N.J., Gilvear D.J. (2008). The spatial dynamics of vertical migration by Microcystis aeruginosa in a eutrophic shallow lake: A case study using high spatial resolution time-series airborne remote sensing. Limnol. Oceanogr..

[53] Gitelson A. (1992). The peak near 700 nm on radiance spectra of algae and water: Relationships of its magnitude and position with chlorophyll concentration. Int. J. Remote Sens..

[54] Kallio K., Koponen S., Pulliainen J. (2003). Feasibility of airborne imaging spectrometry for lake monitoring—A case study of spatial chlorophyll a distribution in two meso-eutrophic lakes. Int. J. Remote Sens..

[55] Moses W.J., Gitelson A.A., Berdnikov S., Povaznyy V. (2009). Estimation of chlorophyll-a concentration in case II waters using MODIS and MERIS data—successes and challenges. Environ. Res. Lett..

[56] Zhang Y., Ma R., Duan H., Loiselle S., Xu J. (2014). A Spectral Decomposition Algorithm for Estimating Chlorophyll-*a* Concentrations in Lake Taihu, China. Remote Sens..

[57] Ouillon S., Douillet P., Petrenko A., Neveux J., Dupouy C., Froidefond J.-M., Andréfouët S., Muñoz-Caravaca A. (2008). Optical Algorithms at Satellite Wavelengths for Total Suspended Matter in Tropical Coastal Waters. Sensors.

[58] Härmä P., Vepsäläinen J., Hannonen T., Pyhälahti T., Kämäri J., Kallio K., Eloheimo K., Koponen S. (2001). Detection of water quality using simulated satellite data and semi-empirical algorithms in Finland. Sci. Total Environ..

[59] Kallio K., Attila J., Härmä P., Koponen S., Pulliainen J., Hyytiäinen U.-M., Pyhälahti T. (2008). Landsat ETM+ images in the estimation of seasonal lake water quality in boreal river basins. Environ. Manag..

[60] Arst H., Erm A., Herlevi A., Kutser T., Leppäranta M., Reinart A., Virta J. (2008). Optical properties of boreal lake waters in Finland and Estonia. Boreal. Env. Res..

[61] Soomets T., Uudeberg K., Jakovels D., Zagars M., Reinart A., Brauns A., Kutser T. (2019). Comparison of lake optical water types derived from Sentinel-2 and Sentinel-3. Remote Sens..

[62] Lins R.C., Martinez J.-M., da Motta Marques D., Cirilo J.A., Fragoso C.R. (2017). Assessment of Chlorophyll-a Remote Sensing Algorithms in a Productive Tropical Estuarine-Lagoon System. Remote Sens..

[63] Pereira-Sandoval M., Urrego E.P., Ruiz-Verdú A., Tenjo C., Delegido J., Soria-Perpinyà X., Vicente E., Soria J., Moreno J. (2019). Calibration and validation of algorithms for the estimation of chlorophyll-a concentration and Secchi depth in inland waters with Sentinel-2. Limnetica.

[64] Toming K., Kutser T., Uiboupin R., Arikas A., Vahter K., Paavel B. (2017). Mapping Water Quality Parameters with Sentinel-3 Ocean and Land Colour Instrument Imagery in the Baltic Sea. Remote Sens..

[65] Kutser T. (2004). Quantitative detection of chlorophyll in cyanobacterial blooms by satellite remote sensing. Limnol. Oceanogr..

[66] Kiefer I., Odermatt D., Anneville O., Wüest A., Bouffard D. (2015). Application of remote sensing for the optimization of in-situ sampling for monitoring of phytoplankton abundance in a large lake. Sci. Total Environ..

[67] Hansen C.H., Burian S.J., Dennison P.E., Williams G.P. (2017). Spatiotemporal Variability of Lake Water Quality in the Context of Remote Sensing Models. Remote Sens..

